# Can stable isotope markers be used to distinguish wild and mass-reared *Anastrepha fraterculus* flies?

**DOI:** 10.1371/journal.pone.0209921

**Published:** 2018-12-31

**Authors:** Victor Botteon, Maria de Lourdes Zamboni Costa, Adalecio Kovaleski, Luiz Antonio Martinelli, Thiago Mastrangelo

**Affiliations:** 1 Centro de Energia Nuclear na Agricultura (CENA), Universidade de São Paulo, Piracicaba, São Paulo, Brazil; 2 Empresa Brasileira de Pesquisa Agropecuária (EMBRAPA), Estação Experimental de Fruticultura de Clima Temperado, Vacaria, Rio Grande do Sul, Brazil; University of Adelaide, AUSTRALIA

## Abstract

The availability of accurate techniques to discriminate between marked laboratory-reared flies and unmarked wild flies captured in monitoring traps is essential for programs that integrate the Sterile Insect Technique (SIT) to manage fruit flies. In this study, the feasibility of using a stable isotope marking technique for the South American fruit fly, *Anastrepha fraterculus* (Wiedemann), was assessed. Wild flies were collected from apple orchards, which are a target of a SIT project in southern Brazil. To verify if adult flies could be labelled by the stable isotopes from larval diets, larvae were reared on two different C_4_-based diets and fruits in laboratory. To evaluate the influence of the two most common attractants applied to capture *A*. *fraterculus* (grape juice and CeraTrap^TM^) and the most common preservation method in fruit fly collections (ethanol), laboratory-reared flies were immersed in McPhail traps containing the respective treatments for two periods of time. Samples were analyzed in an elemental analyzer coupled to a Continuous Flow Isotope Ratio Mass Spectrometer (CF-IRMS) at CENA/USP. The δ^13^C signatures of flies reared on artificial diets differed significantly from the δ^13^C of flies whose larvae were reared on fruits and from wild flies. In contrast, the δ^15^N values were less conclusive and the technique could not rely solely on them. In all cases considered, the *δ*^13^C and *δ*^15^N signatures from males did not differ from females. Despite the alterations caused by the attractants tested and ethanol, laboratory-flies could be distinguished from the wild ones based on *δ*^13^C signatures. This is the first comprehensive study to demonstrate that it is possible to distinguish wild *A*. *fraterculus* from flies reared on larval diets containing C_4_ sugar. The first experimentally derived trophic discrimination factors were also obtained for this species. Thus, intrinsic isotope labelling can serve as a backup to conventional dye marking.

## Introduction

The Sterile Insect Technique (SIT), an autocidal method of control that relies on area-wide inundative releases of sterile insects, has been successfully applied by many operational programs to eradicate, suppress, and contain fruit fly pests [1]. An accurate discrimination between wild and released marked flies captured in monitoring traps is necessary to identify remaining pockets of wild populations, determine actual overflooding ratios, and evaluate progress of the SIT program [2, 3]. In SIT containment programs, it is crucial to avoid misidentification of the released insects, since costly quarantine restrictions could be triggered [2].

The fluorescent dye marking method is still the most used in SIT programs against fruit flies [4]. This method consists of impregnating sterilized pupae with fluorescent dye powder of plant origin (*e*.*g*. Day-Glo^®^). During the emergence from the puparium, the cephalic ptilinum sac of the fly touches the dye, taking some of it when the sac retrieves back inside the head. As fluorescent markings from wings, legs and other body parts of the sterile flies can be lost by weathering, grooming or other reasons, dye marks preferably in the ptilinial suture are sought under a UV lamp or epifluorescence microscope. Despite the high efficiency of the marking process, a very small proportion of the flies can be poorly marked. In the Mexico/United States Mediterranean fruit fly eradication program, for example, the percentage of unmarked captured flies varied between 0.05 and 1% [5]. For flies whose heads do not present internal markings, their reproductive organs must undergo a detailed cytohistological study [6].

Elemental markers can be an alternative to dye marking [7]. Naturally occurring stable isotopes have become a robust identification strategy and its application in SIT programs has been stimulated in recent years [8]. The ratios of carbon and nitrogen stable isotopes (*i*.*e*. ^13^*C*/^12^*C* and ^15^*N*/^14^*N*) have been extensively used by entomologists [9, 10, 11, 12]. Photosynthesis alter the ratio ^13^*C***/**^*12*^*C* in relation to atmospheric CO_2_ depending on the photosynthetic pathways, resulting in different isotopic composition of plants with C_3_ or C_4_ metabolism [13, 14]. On the other hand, the ^15^N**/**^14^N ratio is altered along food chain, with ^15^*N* becoming concentrated as the trophic level increases [10, 12].

As the isotopic signature of an animal reflects the isotopic composition of its food [15, 16], the carbon and nitrogen isotopic signature can be used to track feeding patterns of insects, for instance. Many studies have demonstrated that stable isotopes can be suitable intrinsic markers for mosquitoes [17, 18, 19], tsetse flies [20], moths [21, 22] and fruit flies [23].

The great majority of fruit fly larvae develop on C_3_ plants in the wild [24], but most of the commercially available sugars used in mass-rearing facilities have a C_4_ source, mainly sugarcane (*Saccharum* spp., Poales: Poaceae) [25]. Hood-Nowotny et al. [23] demonstrated that mass-reared Mediterranean fruit flies (medfly), *Ceratitis capitata* (Wiedemann), can be effectively labelled and distinguished from wild populations when C_4_ sugar is used in the larval-rearing diet.

The feasibility of using the stable isotope marking technique for the South American fruit fly, *Anastrepha fraterculus* (Wiedemann), has never been assessed. This is the most economically important fruit fly in commercial apple and peach orchards in southern Brazil [26]. Since 2015, *A*. *fraterculus* populations from this region have been the target of a pest management program called MOSCASUL, which integrates the use of sterile insects and parasitoids [27]. The availability of an alternative accurate labelling technology to identify trapped flies would be extremely valuable for the program, serving as a backup to conventional dye marking.

Aiming to determine whether stable isotopes of carbon and nitrogen would serve as reliable markers for *A*. *fraterculus*, based on natural signature differences between wild and flies reared on artificial diets, the isotopic compositions of larval diets (artificial and natural), adult flies reared under semi-mass rearing conditions, and adults captured from wild populations were assessed. As wild females are usually captured in higher numbers in traps placed in temperate fruit orchards [28], possible differences in carbon and nitrogen isotopic signatures of males and females from both wild and laboratory-reared flies were assessed. The influence of attractive and preservative substances commonly used on traps or for sample preservation over the isotopic signature of the flies was also evaluated.

## Materials and methods

Species identification was performed following Hernández-Ortiz et al. [29]. No endangered or protected species were involved in the study and no specific permits were required for the described field studies.

### Collection of wild *Anastrepha fraterculus* flies

The capture of wild *A*. *fraterculus* flies was carried out in January-February 2017 with McPhail traps containing Ceratrap^TM^ (Bioibérica, Barcelona, Spain). All collections were performed in apple orchards located in the municipality of Vacaria, State of Rio Grande do Sul, Brazil. The flies were captured in three different locations: (a) apple orchard on private property (28°30’59.26” S; 50°52’16.80” W); (b) experimental field area of Embrapa Uva & Vinho (28°30’58.98 S; 50°53’0.97” W); and (c) commercial area of Schio Ltda. (28°32’9.30” S; 50°49’18.30” W).

The traps were inspected at 12 h intervals. For the stable isotope analysis (SIA), 20 flies (10 males and 10 females) from each sampling site were used. All manipulations of flies in the field were carried out using gloves to avoid contaminations. After collection, the flies were gently washed with distilled water and dried for 6 h on paper tissue. The individual samples were conditioned in Eppendorf tubes and stored until the analyses.

### Labelling *Anastrepha fraterculus* flies with different larval diets

To verify if adult flies could be labelled by the stable isotopes from larval diets, an experiment with different artificial diets and fruit hosts was conducted. A bisexual strain of *A*. *fraterculus*, originally established with wild populations from Vacaria [30], has been maintained under semi-mass rearing conditions at the Center for Nuclear Energy in Agriculture of the University of São Paulo (CENA/USP) [31], and it served to provide eggs or adult flies for the test.

In order to obtain the laboratory-reared flies, eggs from the mother colony with the aforementioned bisexual strain were collected and bubbled in water bath at 25°C for 72 h. Aliquots of 2 mL of eggs were seeded in trays containing 1 L of artificial diet, with the larvae remaining there for 10–12 days at 24°C. The larvae were reared on two distinct artificial diets (C_4_-based diets): (a) Diet I (formulated with 50 g of brewer's yeast Brewcell^TM^ (Biorigin, Brazil), 30 g of sugar (sugarcane source) Caravelas^TM^ (Usina Colombo, Brazil), 300 g of corn bran Yoki^TM^ (General Mill Alimentos, Brazil), 2 mL of Nipagin, 2 g of sodium benzoate, 6 g of citric acid, and 1,000 mL of distilled water); and (b) Diet II (a gelled diet adapted from Salles [32], formulated with 3 g of agar, 60 g of corn bran Yoki^TM^ (General Mill Alimentos, Brazil), 60 g of sugar Caravelas^TM^ (Usina Colombo SA, Brazil), 60 g of brewer's yeast Brewcell^TM^ (Biorigin, Brazil), 1 g of sodium benzoate, 6 mL of hydrochloric acid, 8 mL of Nipagin and 900 mL of distilled water). After the larval period, third instar larvae were separated from the artificial diets by washing and then transferred to 500 mL plastic cups with moist vermiculite for pupation in a dark room.

To obtain adult flies from a natural and an alternative host (C_3_-based diets), larvae were reared on apples (*Malus domestica* Borkh. cv. ‘Gala’) [33] and papaya fruits (*Carica papaya* L. cv 'Golden') [34], respectively. The fruits were purchased at the market and cleaned four times with detergent and water. Newly-emerged adults from the mother-colony of the *A*. *fraterculus* bisexual strain were kept in screened cages (30 x 30 x 30 cm; ≈ 600 couples/cage) with water and a mixture of sugar and hydrolyzed brewer's yeast *Bionis* YE MF^TM^ (Biorigin, Lençois Paulista, Brazil) at 3:1 rate and water *ad libitum* [35] under laboratory conditions (25±1°C and 65±10% RH) for the infestation of the fruits. When the flies were 7 days old, the fruits were inserted in each cage separately. After 24 h, the fruits were removed and conditioned in plastic boxes (40 x 20 x 15 cm). Ten days later, the larvae started crawling from the fruits. They were collected twice a day and left to pupate in 500 mL plastic cups with vermiculite.

Treatments were replicated five times with one larval tray or fruit making up each replication. One day before emergence, samples with up to 100 mL of pupae from each replicate were placed in screened cages (30 x 30 x 30 cm) for adult emergence, with only water provided by vials with cotton wicks. After emergence, the 24-h-old flies were frozen to death and the individuals were conditioned in Eppendorf microtubes. Two couples were collected from each of the five replicates of the four treatments. Half of the couples were used for the sex comparison test, while the other half was used to compare the isotopic signals of flies from the different artificial diets and fruits.

### Evaluation of the influence of attractive and preservative substances on the isotopic composition of *A*. *fraterculus* flies

This experiment was carried out to evaluate the influence of the two most commonly attractants applied to capture fruit flies [36] and the most common preservation method in fruit fly taxonomic collections [37] on ^13^C and ^15^N signatures of *A*. *fraterculus* flies. The three substances were: CeraTrap^TM^ (Bioibérica, Barcelona, Spain), concentrated grape juice (Cooperativa Vinícola Garibaldi, Garibaldi, RS, Brazil) and absolute ethanol (Química Moderna Indústria & Comércio, Barueri, SP, Brazil). They were placed separately in McPhail traps (200 mL of each substance per trap), which were distributed randomly in an acclimatized room (25 ± 1°C and 65 ± 10% RH). For the control group, the McPhail traps were filled with distilled water only.

To verify the combined effect of an attractant (CeraTrap^TM^) and ethanol on the isotopic composition of the flies, a group of flies from Diets I and II that had been immersed in CeraTrap^TM^ for seven days was washed and then immersed in absolute ethanol for one week. The treatments and their respective acronyms are listed in S1 Table.

About 100 flies (24-h-old) whose larvae were reared on Diet I and Diet II were separated to be anesthetized by cold (10 min. at -20°C) and then immersed in McPhail traps containing each of the treatments. All the four treatments and control were replicated by three separate traps. Two flies (1 male and 1 female) were randomly collected from each trap on the first and seventh day after immersion, washed with distilled water and dried for 6 h on paper tissue. After that, the samples (*i*.*e*. 6 flies/treatment/day) were individualized as previously described.

### Stable isotope analyses

Analyses of ^13^*C*/^12^*C* and ^15^*N*/^14^*N* ratios were performed to determine the isotopic compositions of wild flies collected in Vacaria, the artificial diets and fruits, newly-emerged laboratory-reared flies, attractive and preservative substances, and fly samples immersed in different substances.

The *δ* was calculated by *δ*X = [(R_sample_/R_standard_) −1], where *δ*X refers to *δ*^13^C or *δ*^15^N, and R is molar ratio of rare to abundant isotopes (^13^*C*/^12^*C* or ^15^*N*/^14^*N*) of the sample (R_sample_) and standard (R_standard_). Isotope ratios are expressed in delta units (parts per thousand; δ ‰) relative to the international reference standards (R_standard_) which are Vienna PeeDee Belemnite (VPDB) and atmospheric nitrogen (N_2_) for *C* and *N*, respectively.

The individualized samples were placed to dry in a ventilated laboratory stove at 50°C for 72 h, and then macerated (whole body) until reaching constant masses. All dried materials were weighed (0.3–0.5 mg for artificial diets, 0.8–1.0 mg for fly samples, and 2.5–2.8 mg for fruit samples) in a precision analytical balance ME 36S (Sartorius, Göttingen, Germany) and enclosed in tin capsules (Elemental Analysis 5 x 3.5 mm). For the ethanol and attractive substances, about 1 mL of the stock substance was used for the analyses.

The enclosed samples were analyzed in an elemental analyzer (CHN 1110 –CE Instruments, Rodano, Italy), coupled to a Continuous Flow Isotope Ratio Mass Spectrometer (CF-IRMS) (Delta Plus–Thermo Scientific, Bremen, Germany). Samples were fired in an oxygen atmosphere at approximately 1700°C, and the resulting N_2_ and CO_2_ passed through a series of scrubbers to remove the impurities and waste water in the elemental analyzer through a chromatographic separation column in ultrapure helium carrier. The CO_2_ and the N_2_ peaks were evaluated in the CF-IRMS to determine the isotopic ratios.

The results were normalized to the international standards using secondary reference materials (NBS-19, NBS-22, IAEA-N1, IAEA-N2) [38]. An internal standard (*i*.*e*. sugarcane leaf) was used for quality control every 11 samples in each run. Each sample was analyzed twice and the analytical error was estimated at ±0.15‰ for both *δ*^13^C and *δ*^15^N measurements.

Trophic discrimination factors (TDFs) of carbon (Δ^13^C) and nitrogen (Δ^15^N) were calculated by subtracting the mean *δ*^13^C or *δ*^15^N values of the whole-body of the 24-h-old flies from the mean *δ*^13^C or *δ*^15^N values of the diet: ΔX = δX_fly’ whole-body_− *δ*X_diet_, where Δ is the trophic discrimination factor and X represents the chemical element being used (^13^C or ^15^N) [39].

### Statistical analyses

The mean values of isotopic compositions (*δ*^13^C and *δ*^15^N) from males and females of the three apple orchards, from each larval diet and fruit were compared by the Student’s *t*-test (α = 0.05). For the *δ*^13^C and *δ*^15^N values from the wild flies of the three collection sites, the one-way analysis of variance *F*-test was applied at the 5% of significance (ANOVA) and, when significant differences were detected, the Tukey’s honestly significance difference (HSD) test (α = 0.05) was applied to compare the means. The mean values of *δ*^13^C and *δ*^15^N from the larval diets, fruits, adults obtained from the diets and wild flies were compared by the Tukey’s test (α = 0.05). For the isotopic compositions (*δ*^13^C and *δ*^15^N) of wild flies and from the different treatments and times (immersion for 1 or 7 days), a two-way analysis of variance *F*-test was applied at a 5% level of significance, considering time and treatment (factorial scheme 2 by 11) as factors. Homogeneity of variances and normality of model residuals were checked in all instances [40, 41]. The analyses were performed by the statistical program SAS 9.4 [42]. Raw isotope data included in S1 Dataset.

## Results

### Isotopic compositions of wild *A*. *fraterculus* flies

A total of 59 wild flies collected in three different apple orchards from Vacaria were analyzed for *δ*^13^C and *δ*^15^N. The isotopic compositions of wild males did not differ from the compositions of females within each of the apple orchards (sites A, B, and C) (*P* > 0.05) (Table 1).

**Table 1:** Isotopic composition of males and females (means ± SE) of wild *Anastrepha fraterculus* flies from three apple orchards.

Orchard	Sex	*δ*^13^C ‰	ANOVA	*δ*^15^N ‰	ANOVA
**A**	Male (n = 10)	-25.5 ± 0.2^†^	F_1,18_ = 3.5; P = 0.079	5.9 ± 0.6	F_1,18_ = 0.81; P = 0.38
	Female (n = 9)	-25.1 ±0.1		5.4 ± 0.3	
**B**	Male (n = 10)	-25.8 ± 0.2	F_1,19_ = 0.47; P = 0.503	4.3 ± 0.6	F_1,19_ = 0.04; P = 0.84
	Female (n = 10)	-25.6 ± 0.3		4.24 ± 0.7	
**C**	Male (n = 10)	-26 ± 0.2	F_1,19_ = 1.0; P = 0.33	5.8 ± 0.7	F_1,19_ = 1.72; P = 0.21
	Female (n = 10)	-26.2 ± 0.2		6.6 ± 0.6	

^†^ Means (± SE) within columns did not differ significantly at the 5% probability level by the Student’s *t*-test.

As the isotopic compositions of the sexes within each collection site did not differ significantly, the *δ*^13^C and *δ*^15^N values from the wild flies of each site were compared among them (Table 2). The *δ*^13^C values of flies from site A differed significantly from the flies from site C, and the *δ*^15^N values of the flies from site B differed from both sites A and C (Table 2). Although there were such differences, the overall δ^13^C mean values of all flies (n = 59) was -25.7 ± 0.2‰, clearly indicating a predominance of C_3_ carbon from apples or natural hosts in the flies. The overall δ^15^N of flies was 5.4 ± 0.6‰.

**Table 2:** Isotopic composition of wild *Anastrepha fraterculus* flies from three different apple orchards.

Orchard	*δ*^13^C ‰	ANOVA	*δ*^15^N ‰	ANOVA
**A** (n = 19)	-25.3 ± 0.2 a^†^	F_2,58_ = 9.45; P< 10^−3^	5.6 ± 0.3 a	F_2,58_ = 10.28;P< 10^−3^
**B** (n = 20)	-25.7 ± 0.1 ab		4.3 ± 0.3 b	
**C** (n = 20)	-26.1 ± 0.1 b		6.2 ± 0.3 a	

^†^ Means (± SE) within columns followed by the same letter do not differ significantly at the 5% probability level by the Tukey’s test.

### Influence of larval diets on the isotopic compositions of *A*.*fraterculus* flies

The isotopic compositions of males and females of flies whose larvae were reared on larval diets and fruits did not differ significantly (P> 0.05) (Table 3).

**Table 3:** Mean isotopic composition of males and females of *Anastrepha fraterculus* flies reared on different larval diets and fruits.

Treatment	Sex	*δ*^13^C ‰	ANOVA	*δ*^15^N ‰	ANOVA
**Flies reared on Diet I**	Male (n = 4)	-15.4 ± 0.1^†^	F_1,7_ = 1.63; P = 0.25	3.95 ± 0.2	F_1,7_ = 0.16; P = 0.71
	Female (n = 4)	-15.2 ± 0.1		4.01 ± 0.06	
**Flies reared on Diet II**	Male (n = 5)	-16 ± 0.2	F_1,9_ = 0.07;P = 0.80	4.3 ± 0.1	F_1,9_ = 0.58; P = 0.467
	Female (n = 5)	-16.1 ± 0.1		4.0 ± 0.3	
**Flies reared on papaya**	Male (n = 5)	-27.1 ± 0.2	F_1,9_ = 0.28; P = 0.615	6.9 ± 0.4	F_1,9_ = 0.04; P = 0.838
	Female (n = 5)	-26.9 ± 0.4		6.8 ± 0.4	
**Flies reared on apple**	Male (n = 4)	-25.8 ± 0.03	F_1,8_ = 0.01; P = 0.93	2.96 ± 0.1	F_1,8_ = 1.48; P = 0.27
	Female (n = 5)	-25.9 ± 0.1		2.9 ± 0.4	

^†^ Means (± SE) within columns did not differ significantly at the 5% probability level by the Student’s *t*-test.

**Fig 1:**
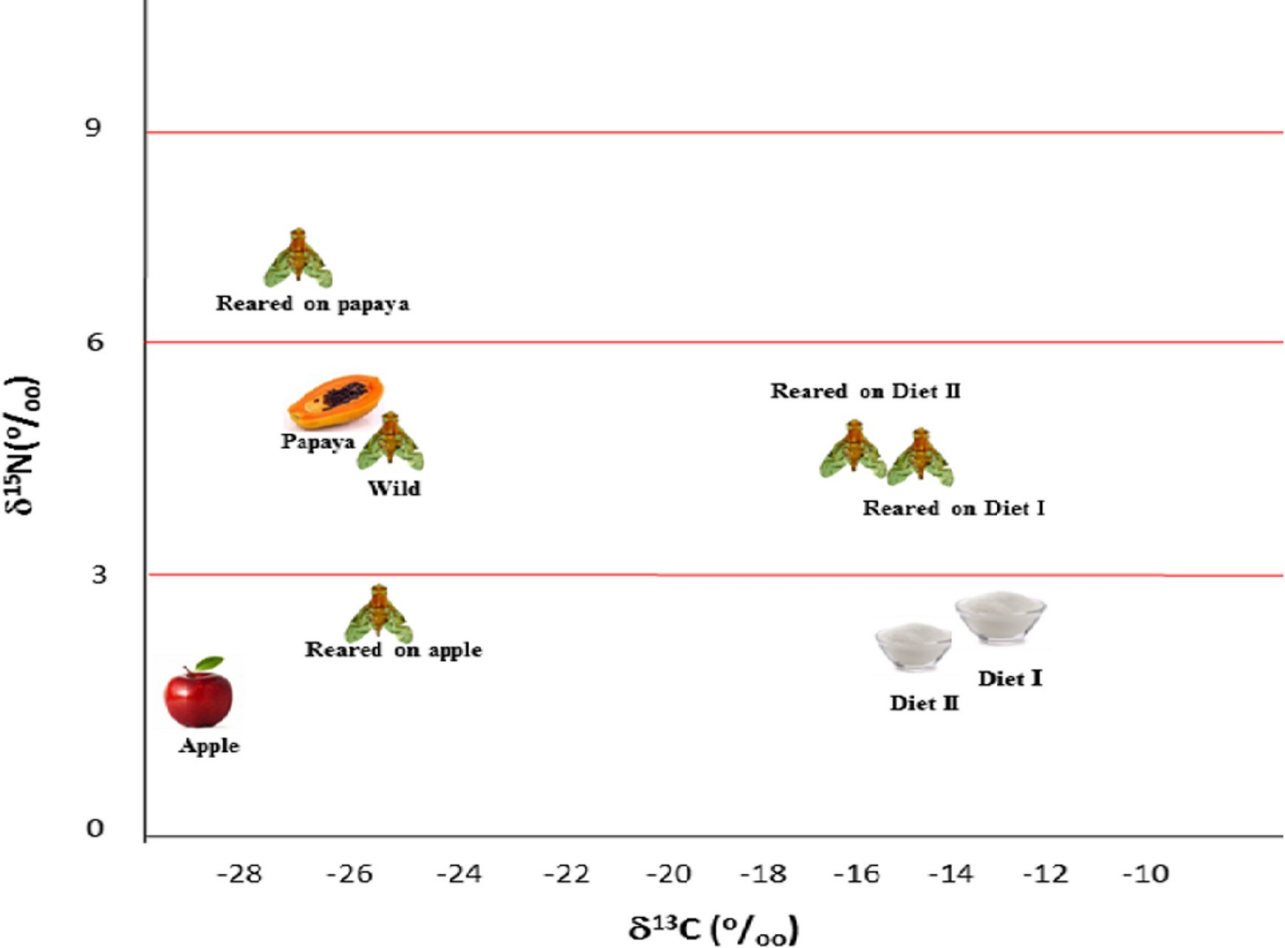
Distribution of the mean values of *δ*^13^C and *δ*^15^N of larval diets, fruits, and *Anastrepha fraterculus* flies reared in laboratory and wild flies (original means±standard errors in Table 4).

The *δ*^13^C and *δ*^15^N of larval diets, fruits, laboratory and wild flies are presented in Table 4 and the distribution of their mean values is shown in Fig 1.

**Table 4:** Isotopic composition of larval diets, fruits, laboratory and wild *Anastrepha fraterculus* flies.

SAMPLE	*δ*^13^C ‰	ANOVA	*δ*^15^N ‰	ANOVA
**Artificial Diet I** (n = 5)	-12.6 ± 0.1 a^†^	F_3,19_ = 14269.6; P< 10^−3^	2.9 ± 0.1 b	F_3,19_ = 96.05; P< 10^−3^
**Artificial Diet II** (n = 5)	-14.3 ± 0.1 b		2.4 ± 0.1 bc	
**Papaya** (n = 5)	-26.0 ± 0.1 c		5.5 ± 0.2 a	
**Apple** (n = 5)	-28.4 ± 0.1 d		2.0 ± 0.2 c	
**Flies reared on Diet I** (n = 10)	-15.3 ± 0.1 a	F_4,42_ = 2063.6; P< 10^−3^	4.0 ± 0.1 c	F_4,42_ = 47.10; P< 10^−3^
**Flies reared on Diet II** (n = 10)	-16.0 ± 0.1 b		4.1 ± 0.2 c	
**Flies reared on papaya** (n = 10)	-27.0 ± 0.2 d		6.8 ± 0.3 a	
**Flies reared on apple** (n = 10)	-25.9 ± 0.1 c		2.9 ± 0.2 d	
**Wild flies** (n = 3) ^‡^	-25.7 ± 0.2 c		5.4 ± 0.6 b	

^†^ Means (± SE) within columns followed by the same letter do not differ significantly at the 5% probability level by the Tukey’s test.

^‡^ The mean values of wild flies from the three different apple orchards were considered.

All diets differed from each other in *δ*^13^C. Considering the *δ*^15^N values, papaya presented the highest *δ*^15^N value (5.5‰), differing significantly from the other diets.

The results also indicated that the *δ*^13^C values of the *A*. *fraterculus* adults reared in laboratory were significantly influenced by the medium in which they developed. The *δ*^13^C mean values of *A*. *fraterculus* reared on artificial Diets I and II reflected that of C_4_-carbon sources, differing from the *δ*^13^C of flies reared on fruits and wild flies. The flies from Diet I presented the highest *δ*^13^C mean value (-15.3‰), while flies from papaya presented the lowest value (-27‰). The *δ*^15^N of *A*. *fraterculus* reared in either fruits or artificial diets differed from the values of wild flies (P < 0.05) (Table 4).

The estimated TDFs showed both enrichment and depletion of *δ*^13^C in adult flies depending on the diet (S2 Table). In comparison to the respective diets, the flies reared on larval Diets I and II had a decrease in *δ*^13^C of 2.7 and 1.7‰, respectively. A decrease of 1‰ in *δ*^13^C value of the flies whose larvae were reared on papaya and an increase of 2.5‰ in *δ*^13^C of the flies whose larvae were reared on apple were also observed.

Considering the Δ ^15^N values, only *δ*^15^N enrichment was observed in all treatments, which is expected with the increase of trophic level [10, 43] (S2 Table).

### Influence of capture and preservative substances on the isotopic composition of *A*. *fraterculus* flies

The mean *δ*^13^C and *δ*^15^N values of CeraTrap^TM^ were -25 ± 0.1‰ and 3.4 ± 0.2‰, respectively. For grape juice, the means were -26.3 ± 0.1‰ and 2.8 ± 0.1‰, respectively; while the values from absolute ethanol were -12.1 ± 0.5‰ for *δ*^13^C and 1.7‰ for *δ*^15^N. For wild flies and those from control treatments (Water I and II; acronyms for the treatments are given in Tables 5 and 6), the isotopic composition values were kept without noticeable variation until the seventh day of evaluation.

**Table 5:** Mean δ^13^C values of wild and laboratory *Anastrepha fraterculus* flies that were immersed in attractive and preservative substances for one and 7 days.

TREATMENT	*δ*^13^C ‰ (1 day)	*δ*^13^C ‰ (7 days)	ANOVA
**Wild flies** (n = 3) ^†^	-25.7 ± 0.2 A	-25.7 ± 0.2 A	-
**Water I** (n = 6)	-15.4 ± 0.1 DE	-15.4 ± 0.1 DE	-
**Water II** (n = 6)	-15.9 ± 0.1 CD	-15.9 ± 0.1 CDE	-
**ET I** (n = 6)	-15 ± 0.2 E a ^‡^ ^§^	-15 ± 0.2 E a	F_1,11_ = 0.03; P = 0.856
**ET II** (n = 6)	-16.8 ± 0.1 B a	-17.1 ± 0.1 BCD b	F_1,11_ = 6.53; P = 0.028
**CT I** (n = 6)	-15.2 ± 0.2 DE a	-16.3 ± 0.3 CDE b	F_1,11_ = 11.14; P = 0.007
**CT II** (n = 6)	-15.6 ± 0.2 CDE a	-16.8 ± 0.2 BCD b	F_1,11_ = 13.82; P = 0.004
**GJ I** (n = 6)	-16.4 ± 0.1 BC a	-17.3 ± 0.8 BC a	F_1,11_ = 1.39; P = 0.266
**GJ II** (n = 6)	-16.8 ± 0.1 B a	-18.1 ± 0.9 B a	F_1,11_ = 2.47; P = 0.147
**CTET I** (n = 6)	-16.3 ± 0.3 BC a	-16.3 ± 0.1 CDE a	F_1,11_ = 0,01; P = 0.963
**CTET II** (n = 6)	-16.8 ± 0.2 B a	-17.6 ± 0.2 BC b	F_1,11_ = 6.2; P = 0.03
**ANOVA**	F_10,62_ = 399.13; P<10^−3^	F_10,62_ = 80.03; P< 10^−3^	

^†^ The mean values of wild flies from the three different apple orchards were considered all together. Acronyms: ET = ethanol; CT = CeraTrap^TM^; GJ = grape juice; CTET = CeraTrap^TM^ for 7 days and then flies immersed in absolute ethanol for 7 days; I = performed with flies from Diet I; II = performed with flies from Diet II.

^‡^ Means (± SE) followed by the same uppercase letter in the columns do not differ significantly at the 5% probability level by the Tukey’s test.

^§^ Means (± SE) followed by the same lowercase letter in the lines do not differ significantly at the 5% probability level by the Student’s *t-*test.

**Table 6:** Mean *δ*^15^N values of wild and laboratory *Anastrepha fraterculus* flies that were immersed in attractive and preservative substances for one and 7 days.

TREATMENT	*δ*^15^N ‰ (1 day)	*δ*^15^N ‰ (7 days)	ANOVA
**Wild flies** (n = 3) ^†^	5.4 ± 0.6 AB	5.4 ± 0.6 AB	-
**Water I** (n = 6)	4 ± 0.1 C	4 ± 0.1 C	-
**Water II** (n = 6)	4.1 ± 0.3 C	4.1 ± 0.3 C	-
**ET I** (n = 6)	4.4 ± 0.1 BC a ^‡^ ^§^	4.5 ± 0.1 BC a	F_1,11_ = 0.28; P = 0.608
**ET II** (n = 6)	4.6 ± 0.1 BC a	4.9 ± 0.1 BC b	F_1,11_ = 15.25; P = 0.002
**CT I** (n = 6)	4.5 ± 0.2 BC a	6.3 ± 0.4 A b	F_1,11_ = 18.35; P = 0.001
**CT II** (n = 6)	4.4 ± 0.2 BC a	5.5 ± 0.2 AB b	F_1,11_ = 13.59; P = 0.004
**GJ I** (n = 6)	4.8 ± 0.3 BC a	4.2 ± 0.4 C a	F_1,11_ = 1.71; P = 0.22
**GJ II** (n = 6)	4.6 ± 0.1 BC a	4.8 ± 0.1 BC a	F_1,11_ = 0.99; P = 0.343
**CTET I** (n = 6)	6.3 ± 0.4 A a	5.4 ± 0.1 AB b	F_1,11_ = 5.86; P = 0.036
**CTET II** (n = 6)	5.5 ± 0.2 AB a	5 ± 0.2 BC a	F_1,11_ = 2.01; P = 0.187
**ANOVA**	F_10,62_ = 8.54; P< 10^−3^	F_10,62_ = 8.35; P< 10^−3^	

^†^ The mean values of wild flies from the three different apple orchards were considered all together. Acronyms: ET = ethanol; CT = CeraTrap^TM^; GJ = grape juice; CTET = CeraTrap^TM^ for 7 days and then flies immersed in absolute ethanol for 7 days; I = performed with flies from Diet I; II = performed with flies from Diet II.

^‡^ Means (± SE) followed by the same uppercase letter in the columns do not differ significantly at the 5% probability level by the Tukey’s test.

^§^ Means (± SE) followed by the same lowercase letter in the lines do not differ significantly at the 5% probability level by the Student’s *t-*test.

The *δ*^13^C and *δ*^15^N values of the flies were significantly affected after immersion in different substances (F = 271.83, *P*< 10^−3^; F = 11.93, *P* < 10^−3^, respectively), but the exposure time (1 or 7 days) in traps was significant only for *δ*^13^C (F = 17.49, *P*< 10^−3^, F = 1.81, *P* = 0.18, respectively). The interaction of factors time and treatments was not significant on the *δ*^13^C values of the flies (F = 1.90, *P* = 0.051; F = 4.96, *P*< 10^−3^, respectively).

Grape juice caused the most of *δ*^13^C depletion (up to 0.6‰ and 2.1‰ after one and 7 days of immersion, respectively). Consequently, the flies immersed in grape juice for 7 days differed significantly from both water controls (*P* < 0.05) (Table 5). After 7 days of immersion, most of the treatments resulted in depletion of *δ*^13^C compared to the Water I control (CT I = -0.9‰; GJ I = -1.9‰), with enrichment only in ET I (+0.4‰). Compared to the Water II control, depletion was observed in all treatments (ET II = -1.2‰; CT II = -0.9‰; GJ II = -1.9‰).

In its turn, the flies immersed in CeraTrap^TM^ for 7 days had their *δ*^13^C values not significantly altered compared to controls, but CeraTrap^TM^ was the substance that most impacted the *δ*^15^N values (Table 6). Actually, all the substances tested enriched *δ*^15^N values after 7 days in comparison to Water I (ET I = +0.5‰; CT I = +2.3‰; GJ I = +0.1‰) and Water II controls (ET II = +0.8‰; CT II = +1.4‰; GJ II = +0.7‰). When CeraTrap^TM^ and ethanol were combined, differences by up to -1.7 and +1.4‰ in *δ*^13^C and *δ*^15^N signatures were respectively observed (Table 6).

Considering factor time within each of the treatments, significant differences in either *δ*^13^C and *δ*^15^N values from the ET II, CT I and CT II treatments were observed (*P*<0.05) (Tables 5 and 6). On the seventh day of evaluation, the *δ*^13^C values of the flies from these three treatments suffered depletions of 0.3‰, 1.1‰ and 1.2‰, while *δ*^15^N was enriched in 0.3‰, 1.8‰ and 1.1‰, respectively.

Independent of treatment and immersion time, the differences in *δ*^13^C values between wild flies and those reared on Diets I and II were still significant (*P* < 0.05) (Table 5). Considering the *δ*^15^N signals, the flies from the different treatments (except the controls and GJ I after 7 days) did not differ significantly from the wild flies (Table 6).

## Discussion

Significant differences in *δ*^13^C values were observed when larvae fed on diets with distinct isotope signatures (Table 4). Due to the amounts of cane sugar and corn used in Diets I and II, they can be considered C_4_-based diets. The *δ*^13^C signals of these diets ranged from -12.5 to -14.5‰, but some C_3_ ingredients such as brewer’s yeast might have helped to slightly decrease the signatures from both diets [44]. The *δ*^13^C signatures of *A*. *fraterculus* reared on artificial diets tended to reflect those of the larval diets (-15.3‰ and -16‰, respectively), differing from the mean *δ*^13^C values from flies whose larvae were reared on fruits (apple and papaya) and wild flies (Table 4).

In Brazil, 116 hosts of *A*. *fraterculus* are known [45, 46, 47, 48], with most of the species belonging to the families Myrtaceae and Rosaceae [46]. In the State of Rio Grande do Sul, besides apple orchards, sources of infestation are observed in commercial peach orchards (*Prunus persica*), citrus (*Citrus* spp.) and grapevine (*Vitis vinifera*) [45, 46, 48, 49, 50]. Cultivation of new fruits, such as blackberry (*Rubus* spp.), blueberry (*Vaccinium ashei*) and Brazilian cherry (*Eugenia uniflora*), may also contribute with new breeding sites for *A*. *fraterculus* [51]. In a recent study performed by Marsaro Junior [52], infestations by *A*. *fraterculus* were observed in fruits of *Annona rugulosa*, *Acca sellowiana*, *Campomanesia guazumifolia*, *Campomanesia xanthocarpa*, *Diospyros kaki*, *Eugenia involucrata*, *Eugenia pyriformis*, *Eugenia uniflora*, *Psidium cattleianum*, *Psidium guajava* and *Prunus persica*. In the municipality of Vacaria, large infestations of this pest can be observed in *Campomanesia xanthocarpa*, *Eugenia involucrata* and *Feijoa sellowiana* [53]. All these fruits follow the C_3_ photosynthetic pathway [13] and future determinations of their isotopic signatures could help to elucidate the differences observed among wild flies from different sites. Although some variability in their carbon isotopic composition will exist, their δ^13^C values will be consistently lower than flies reared on C_4_-based diets (Fig 1). This fact assures the usefulness of stable isotopes to label mass-reared *A*. *fraterculus*.

This usefulness is reinforced by the fact that the *δ*^13^C values of males did not differ from females, neither in flies reared on different larval diets nor in wild flies (Tables 1 and 3). This finding is in line with Hood-Nowotny et al. [22] that also found no significant differences in isotope signatures between males and females of the cactus moth. However, it seems that sex differences depend on the species. For instance, Hood-Nowotny et al. [23] observed differences in the *δ*^13^C signals between males and females of *C*. *capitata* reared on artificial larval diets, and Sato and Azuma [43] detected significant differences in δ^13^C of males and females of *Euthrix potatoria* L. However, such differences were not large enough to preclude the use of stable isotope as markers of flies reared in laboratory [23]. Future analyses of specific body structures instead of whole body could help to elucidate the existence of any differences in isotopic signatures between sexes of *A*. *fraterculus*.

Since aggregation of isotopes from other sources or loss of molecules carrying lighter isotopes can result in improper interpretation of isotopic signatures [54], knowledge of the impact of attractant and preservation methodologies on isotopic values of insect samples is essential. For example, fuel and commercial ethanol caused enrichments in *δ* ^13^C and *δ* ^15^N of cricket samples as great as 1.5 and 0.9‰, respectively [55]. Hogsden and McHugh [56] compared that ethanol-preserved and lipid-extracted samples of 10 common New Zealand stream invertebrates enriched by 1–2‰ in *δ* ^13^C to frozen samples and found that short-term storage in 70% ethanol caused effects comparable to lipid removal. According to these studies, samples subjected to organic solvents can lose dissolved lipids or gain solvent constituent carbon, which can enhance the ^13^*C* signal.

Our results corroborate the findings of previous studies, demonstrating that ^13^*C* and ^15^*N* signatures of fly specimens can be affected by the attractant or preservative substance used and time that flies remain in a trap (Tables 5 and 6). Despite the alterations produced by the attractants tested and ethanol, it was still possible to distinguish laboratory-flies from the wild ones based on the *δ*^13^C values at the 5% probability level of significance (Table 5). As field traps are surveyed on a weekly basis [26], the use of CeraTrap^TM^ and grape juice would not compromise the isotope-based method developed in this study. However, the effects of longer storage periods (> 30–60 days) in ethanol should be further investigated to confirm if the fly samples would still show reliable SIA results.

In contrast, the *δ*^15^N values were less conclusive compared to *δ*^13^C, with no significant differences among some treatments, making it impossible to distinguish the flies reared on artificial diets from wild ones. Compared to the respective diets, the flies reared on larval Diets I and II had an increase in *δ*^15^N values of 2.1‰ and 1.8‰, respectively (S2 Table). Flies can produce proteins that are enriched in ^15^*N* relative to the food source and periods of starvation can also induce enrichment in some tissues [57, 58]. The fractionation of stable isotopes of nitrogen is more unpredictable than for stable isotopes of carbon [9, 10], and, therefore, traceability should not rely solely on *δ*^15^N signals, unless the artificial diet is enriched with *N*-sources [19].

This study provided the first experimentally derived TDFs of stable carbon and nitrogen isotopes for *A*. *fraterculus*. Overall, the Δ^13^C values ranged from -2.7 to 2.5‰, while Δ^15^N varied from 0.9 to 2.1‰ (S2 Table). The isotopic enrichments or depletions observed are consistent with published data for other species. In a study involving 22 terrestrial herbivorous arthropods feeding on 18 different host plants, Spence and Rosenheim [59] verified TDFs from plant to herbivores in the range of -3.5‰ to 1.9‰ in *δ*^13^C (-0.5±0.3‰ of average) and *δ*^15^N enrichments of -0.2‰ to 6.6‰ (1.9±0.8‰ of average). Markow et al. [60] reported differences in *δ*^13^C and *δ*^15^N between wild-caught *Drosophila* spp. and their respective natural hosts between -1.8 and 5.2‰ and -0.4 and 5.0‰, respectively. In mass-reared medflies from Madeira Archipelago and Guatemala, the Δ^13^C values ranged from -1.7 to 2.1‰ and Δ^15^N ranged from 2.9 to 3.7‰ [23]. Intraspecific variations in TDFs are common and can be expected depending on diet quality, developmental stage and nutritional status of the consumer, different metabolic rates in animal tissues, lipid extraction and other factors [57, 61, 62, 63].

In conclusion, this is the first comprehensive study to demonstrate that it is possible to distinguish wild *A*. *fraterculus* from flies reared on larval diets containing C_4_ sugars. We provide a simple but effective marking method, with the advantage of not requiring changes in the larval rearing protocols. With recent technical advances in mass spectroscopy, the costs of isotopic analyses are now comparable to the most common molecular and chemical analyses (even < US$ 5–15.00/sample).

Our isotope marking system would have a wide application in SIT projects against *A*. *fraterculus*. For example, sterile flies are released in forested areas surrounding apple and peach orchards at southern Brazil, but occasionally some of them get caught in traps inside commercial orchards. If the flies are unmarked and a cytohistological analysis of their testis or ovaries is not possible to be performed (*e*.*g*. females cannot be easily distinguished on the basis of ovary development when they are younger than 7 days) [64], the SIA could be used to determine if the flies are wild, resolving the doubts of farmers and SIT managers. In addition to the already raised issues that should be further investigated, the next step of our research will be to determine isotopic turnover rates and the persistence of the stable isotopes of *C* and *N* during the life of *A*. *fraterculus* when the adult diet changes from C_4_ to C_3_ food sources in nature.

## Supporting information

S1 Dataset Dataset containing the raw isotope data collected for Tables 1 to 6. (XLSX)

S1 Table Treatments used for the evaluation of the influence of attractive and preservative substances on the isotopic composition of *Anastrepha fraterculus* flies (acronyms in parentheses). (DOCX)

S2 Table Mean trophic discrimination factors (Δ) between *Anastrepha fraterculus* flies and their respective larval diets. (DOCX)
