# Supplementary material for: Can stable isotope markers be used to distinguish wild and mass-reared Anastrepha fraterculus flies?
Source: PLoS One. 2018 Dec 31;13(12):e0209921. doi: 10.1371/journal.pone.0209921 (PMC6312238; doi:10.1371/journal.pone.0209921)
Supplement: S1 Table — (DOCX) [file pone.0209921.s002.docx]

**S1 Table. Treatments used for the evaluation of the influence of attractive and preservative substances on the isotopic composition of *Anastrepha fraterculus* flies (acronyms in parentheses).**

| **Treatments with flies reared on Diet I** | **Treatments with flies reared on Diet II** |
| --- | --- |
| Distilled water (Water I) | Distilled water (Water II) |
| Absolute ethanol (ET I) | Absolute ethanol (ET II) |
| Ceratrap^TM^ (CT I) | Ceratrap^TM^ (CT II) |
| Grape Juice (GJ I) | Grape Juice (GJ II) |
| Ceratrap^TM^ to Ethanol (CTET I) | Ceratrap^TM^ to Ethanol (CTET II) |

Acronyms: ET= ethanol; CT= CeraTrap^TM^; GJ= grape juice; CTET= CeraTrap^TM^ for 7 days and then flies immersed in absolute ethanol for 7 days; I= performed with flies from Diet I; II= performed with flies from Diet II.
