# Supplementary material for: Can stable isotope markers be used to distinguish wild and mass-reared Anastrepha fraterculus flies?
Source: PLoS One. 2018 Dec 31;13(12):e0209921. doi: 10.1371/journal.pone.0209921 (PMC6312238; doi:10.1371/journal.pone.0209921)
Supplement: S2 Table — (DOCX) [file pone.0209921.s003.docx]

**S2 Table. Mean trophic discrimination factors (Δ) between *Anastrepha fraterculus* flies and their respective larval diets.**

| **Treatment** | Δ**^13^C ‰** | Δ**^15^N ‰** |
| --- | --- | --- |
| **Diet I** | -2.7 | 2.1 |
| **Diet II** | -1.7 | 1.8 |
| **Apple** | 2.5 | 0.9 |
| **Papaya** | -1.0 | 1.3 |
